# The influence of fish consumption on serum n-3 polyunsaturated fatty acid (PUFA) concentrations in women of childbearing age: a randomised controlled trial (the iFish Study)

**DOI:** 10.1007/s00394-020-02326-w

**Published:** 2020-07-28

**Authors:** Marie C. Conway, Emeir M. McSorley, Maria S. Mulhern, Toni Spence, Edwin van Wijngaarden, Gene E. Watson, Karin Wahlberg, Daniela Pineda, Karin Broberg, Barry W. Hyland, Diego F. Cobice, J. J. Strain, Alison J. Yeates

**Affiliations:** 1 Nutrition Innovation Centre for Food and Health (NICHE), School of Biomedical Sciences, Ulster University, Cromore Road, Coleraine, BT52 1SA Northern Ireland UK; 2 School of Medicine and Dentistry, University of Rochester, Rochester, USA; 3 The Laboratory of Medicine, Division of Occupational and Environmental Medicine, Lund University, Lund, Sweden; 4 Institute of Environmental Medicine, Karolinska Institutet, Stockholm, Sweden; 5 Mass Spectrometry Centre, Biomedical Sciences Research Institute (BMSRI), Ulster University, Coleraine, Northern Ireland UK

**Keywords:** Fish consumption, Polyunsaturated fatty acids, Eicosapentaenoic acid, Docosahexaenoic acid, Fatty acid desaturase, FADS

## Abstract

**Purpose:**

Long-chain polyunsaturated fatty acids (LCPUFA) can be synthesised endogenously from linoleic acid (LA) and α-linolenic acid (ALA) in a pathway involving the fatty acid desaturase (FADS) genes. Endogenous synthesis is inefficient; therefore, dietary intake of preformed LCPUFA from their richest source of fish is preferred. This study investigated the effect of fish consumption on PUFA concentrations in women of childbearing age while stratifying by FADS genotype. The influence of fish consumption on lipid profile, and markers of inflammation and oxidative stress was also examined.

**Methods:**

Healthy women (*n* = 49) provided a buccal swab which was analysed for *FADS2* genotype (rs3834458; T/deletion). Participants were stratified according to genotype and randomised to an intervention group to receive either no fish (*n* = 18), 1 portion (*n* = 14) or 2 portions (*n* = 17) (140 g per portion) of fish per week for a period of 8 weeks. Serum PUFA was analysed at baseline and post-intervention. Lipid profile, and markers of inflammation and oxidative stress were also analysed.

**Results:**

Participants consuming 2 portions of fish per week had significantly higher concentrations of eicosapentaenoic acid (EPA), docosahexaenoic acid (DHA) and total n-3 PUFA, and a lower n-6:n-3 ratio compared to those in the no fish or 1 portion per week group (all *p* < 0.05). Fish consumption did not have a significant effect on biomarkers of oxidative stress, inflammation and lipid profile in the current study.

**Conclusion:**

Consumption of 2 portions of fish per week has beneficial effects on biological n-3 PUFA concentrations in women of childbearing age; however, no effects on oxidative stress, inflammation or lipid profile were observed. This trial was registered at www.clinicaltrials.gov (NCT03765580), registered December 2018.

**Electronic supplementary material:**

The online version of this article (10.1007/s00394-020-02326-w) contains supplementary material, which is available to authorized users.

## Introduction

Polyunsaturated fatty acids (PUFA) have important roles in health, including a role in the inflammatory response, cell membrane structure and function, and are particularly important during pregnancy [1]. Arachidonic acid (AA) and docosahexaenoic acid (DHA) are essential for brain development [2–4], particularly during the third trimester of pregnancy when DHA accumulation in the brain is most rapid [5, 6]. Higher PUFA status in pregnant women has also been linked to improved child cognitive outcomes [7–12], and has been found to have a role in reducing inflammation, oxidative stress and lipid profile [13–15].

Linoleic acid (LA) and α-linolenic acid (ALA) are essential fatty acids which cannot be synthesised in vivo and must be provided by the diet [16]. These precursor molecules go through a series of elongation and desaturation steps to produce long-chain (LC) PUFA including n-6 AA, and eicosapentaenoic acid (EPA) and DHA of the n-3 family. The fatty acid desaturase 1 (*FADS1)* and *FADS2* genes are located on chromosome 11q12–q13.1, and encode the Δ-5 and Δ-6 desaturase enzymes (D5D and D6D respectively) involved in the elongation and desaturation pathway [17]. Single nucleotide polymorphisms (SNPs) in *FADS1* and *FADS2* have been reported to influence LCPUFA status, with minor allele carriers having lower concentrations of DHA, EPA and AA [18–23]. Even in individuals with high LCPUFA intake, with increasing number of minor alleles of *FADS2* rs3834458, the concentrations of AA were significantly decreased [18]. This SNP is in strong linkage disequilibrium [24] with other FADS SNPs, e.g. rs174537 and rs174545, that have also been linked to LCPUFA status [18, 20, 22, 23]. Thus, rs3834458 tags functional variation of the FADS region. The endogenous synthesis pathway, however, is inefficient [25], and thus, dietary intake of preformed LCPUFA is preferred to meet requirements.

Fish is a rich source of many nutrients including PUFA. Fish consumption in the UK is below the current recommendations of two portions of fish per week [26]. Increased fish consumption is hypothesised to improve biological status of LCPUFA [27]; nevertheless, there are conflicting findings with regard to LCPUFA dietary intake from fish and biological LCPUFA status. Increased fish consumption has been found to be associated with increased PUFA status in pregnant women [28, 29], and non-pregnant, adult populations [30, 31]. Research to date has not conclusively shown an increase in LCPUFA status following increased dietary intake, with some researchers reporting no association between fish consumption and LCPUFA status [9, 32, 33]. Studies have focused on fish consumption of pregnant women [9], and in mothers and their newborns [33, 34] in observational and intervention studies, respectively. The observational study by Bonham et al*.* [9] may have lacked associations owing to physiological factors of pregnancy, such as the transfer of PUFA from mother to foetus. An observational study in non-pregnant adults concluded associations between fish consumption and biological PUFA status cannot be generalised to all fish intakes, with fatty fish influencing biological LCPUFA status; whereas, the consumption of lean fish does not. The authors suggest these findings may be owing to EPA and DHA being metabolised into plasma differently [32]. It is also important to acknowledge that factors other than diet are associated with PUFA status, such as sex, physical activity and genetics [35]. During pregnancy, the mobilisation of DHA into circulation may alter erythrocyte PUFA profile, and subsequently lead to a lower correlation between circulating PUFA status and dietary intake [29].

The evidence available to date for associations between fish consumption and PUFA status is mainly observational [9, 27–32]. There is a lack of studies intervening with fish consumption to investigate whether consumption will increase biological PUFA status. In an intervention study in pregnant women who were low habitual consumers of fish, women were randomised to consume either 2 portions of salmon per week or to continue their habitual diet [34]. This study concluded that the consumption of 2 portions of salmon per week will increase EPA and DHA biological status in pregnant women who do not regularly consume oily fish. The role of genetics has not been controlled for in studies completed to date, and thus, FADS SNPs may account in part for the discrepancies in the influence of fish consumption on PUFA status. The conversion of the precursors LA and ALA to LCPUFA may be greater in those consuming less fish [36]; therefore, given the influence of the FADS genotype on the endogenous synthesis pathway, it is possible that individuals with the minor allele for FADS may benefit from direct consumption of preformed EPA and DHA. Research in women of childbearing age is of importance owing to the key role of PUFA during pregnancy and for child development.

To our knowledge, no randomised controlled trials (RCTs) intervening with fish have considered confounding by FADS genotype. Therefore, the primary aim of the current study was to investigate whether fish consumption influences serum n-3 PUFA concentrations in women of childbearing age while stratifying by FADS genotype. It was hypothesised that fish consumption will increase concentrations of n-3 LCPUFA in a dose–response manner when allowing for a major confounding factor of genetic variation in *FADS2* (rs3834458, T/deletion). Fish consumption has been shown to decrease biomarkers of inflammation [37], oxidative stress [38] and lipid profile [39]. The effect of fish intervention on these markers was also assessed in the current study in secondary analysis. The influence of fish consumption on serum n-3 PUFA concentrations within genotype groups for carriers of the rs3834458 (TT) and for carriers of the minor allele (Tdel or deldel) was also investigated in secondary analysis.

## Methods

### Study design and population

The “iFish Study” (registered at www.clinicaltrials.gov (NCT03765580)) was an 8-week RCT with the overall aim of investigating whether fish consumption influences PUFA status in women of childbearing age when accounting for FADS genotype. Female participants were recruited within Ulster University, Coleraine, UK and the surrounding area from October 2016. All those interested in taking part in the study completed a screening questionnaire. Inclusion criteria were: being a healthy female of childbearing age (aged between 18 and 45 years), premenopausal, and not planning on becoming pregnant during the course of the study. Furthermore, suitable participants had to be low consumers of fish (< 2 portions of fish per week), willing to consume 1, 2 or no portions of fish per week, non-consumers of fish oil or protein supplements, and not allergic to seafood. Exclusion criteria included: being a regular consumer of fish and not willing to do washout period where they reduced fish consumption in advance of the study; allergic to seafood, taking fish oil or protein supplements, being pregnant or menopausal, or having very short hair. All participants provided written informed consent and the study was approved by Ulster University Research Ethics Committee (REC/16/0077). All research was conducted in accordance with the 1964 Declaration of Helsinki and its amendments.

### SNP selection and genotype determination

Eligible participants provided a buccal swab. Buccal swab samples were stored at -80 °C and shipped to Lund University, Sweden for DNA extractions and genotyping. DNA was isolated using the QIAamp DNA Mini Kit (Qiagen, Hilden, Germany) according to the manufacturer’s protocol. Rs3834458 was genotyped on an ABI 7900HT Fast Real Time PCR System (Applied Biosystems, Thermo Fisher, Waltham, Massachusetts, USA) using a custom TaqMan assay (Thermo Fisher Scientific) according to the manufacturer’s instructions. As mentioned, rs3834458 was selected based on its strong associations with PUFA status [18, 40]. This SNP is referred to as an intronic variant and has a minor allele frequency (MAF) of 35% in European populations [24]. As a quality control measure, approximately 5% of samples were randomly selected and reanalysed. There was 100% agreement between original and duplicate samples. Further quality assessment of the data was completed by evaluating Hardy–Weinberg equilibrium using Chi-Square test.

### Recruitment and randomisation

Recruitment of participants involved the distribution of information emails, posters and leaflets within Ulster University, Coleraine, UK and the surrounding area. A total of 124 women expressed interest in the study while 89 completed the screening questionnaire, of which 66 were deemed eligible and were recruited. Power calculations were based on observing a difference in PUFA status with an effect size of 1.8 [41] in a within–between ANOVA using G-Power with a power level of 80% and significance level of 0.05. It was determined that a total of 10 people were required for each of the three intervention groups (no fish, 1 portion and 2 portions of fish per week). Participants were stratified based on rs3834458 genotype and randomised into an intervention group of either no fish, 1 portion or 2 portions of fish per week by an independent Clinical Trials Manager using MINIM software (MINIM, UK). Of the eligible participants randomised after genotyping, 17 individuals did not commence the study (*n* = 7 did not wish to take part any longer; and *n* = 10 were not contactable to arrange a baseline appointment). These 17 individuals were equally distributed across the intervention groups and there were no significant differences in age or fish consumption between those who took part and those who did not commence the study. Forty-nine apparently healthy females began the study.

### Intervention period

Participants were invited to attend two sampling appointments at the Human Intervention Study Unit (HISU) at Ulster University at baseline and post-intervention beginning in January 2017. At each time point, participants provided biological samples including blood, and information on habitual dietary intake, anthropometric measurements and general health and lifestyle information was also recorded. Study participants were provided with a lunch dish either once or twice a week (depending on their intervention group) in the HISU. Each lunch contained a 140-g portion of fish according to the intervention group to which they were randomised (either tuna or sardines for the duration of the study). Those randomised to the no fish group also received a lunchtime meal with no fish once per week. Participants were given the option of choosing between a salad, baked potato or sandwich lunch. Each lunchtime option was entered on Nutritics dietary analysis software (Nutritics Ltd, Swords, Dublin) to calculate energy and macronutrient composition to ensure similar amounts were provided across each of the intervention groups. Leftovers were weighed and recorded to measure compliance. The same batch numbers were used throughout the study for the tinned tuna and sardines, and samples of the fish were analysed for PUFA concentrations (ALS Life Sciences, UK) prior to the study commencing. Total n-3 concentrations (g/100 g) were 4.57 for tuna, and 6.47 for sardines.

### Blood sampling and anthropometric measurements

Fasting blood samples collected at baseline and post-intervention were processed to obtain serum and plasma by centrifugation at 3500 rpm for 15 min at 4 °C. An aliquot of plasma had 0.005% butylated hydroxytoluene (BHT) added as an antioxidant at the time of blood processing. All aliquots were subsequently frozen and stored at  – 80 °C until batch analysis. Body weight (to the nearest 0.1 kg) was measured using TANITA digital scales (TANITA Europe, The Netherlands). Height was measured to the nearest centimetre using a stadiometer and body mass index (BMI, kg/m^2^) was then calculated.

### PUFA analysis

Serum samples were analysed for total PUFA, using a method adapted from Folch et al*.* [42]. This method involved extraction of total lipids followed by methylation to fatty acid methyl esters (FAME) using boron trifluoride methanol (BF_3_) (Sigma Aldrich, UK) [43]. FAME were quantified using gas chromatography mass spectrometry (GC–MS) (7890A-5975C; Agilent Technologies UK Ltd, UK). Analysis was completed in split mode, with a BPX70 capillary GC column (SGE Analytical Science) (length 30 m, internal diameter 250 µm and film thickness 0.25 µm), using helium as the carrier gas (constant flow at 1.0 ml/min; purity 99.9999%). Samples were injected using an automatic liquid sampler (ALS) (injection volume 1 µl) at a temperature of 130 °C; which was then ramped at 15 °C/min to 200 °C and then at 30 °C/min to 250 °C where it was held for 5 min. Mass spectrometry was operated in positive ion mode using an electron ionisation (EI) source. Mass range was set to 50–500 Da and acquisition was performed by total ion chromatogram (TIC). LA, ALA, AA, EPA and DHA were identified by their retention time and corresponding qualifier ions with reference to those of commercially available fatty acid standards (Sigma Aldrich, UK), and were quantified by use of an internal standard, heptadecanoic acid (C17:0) (Sigma Aldrich, UK) and corresponding PUFA target ions (quantifiers). For the current study, total n-6 (mg/ml) was calculated by summing LA and AA concentrations, and ALA, EPA and DHA were summed to calculate total n-3 (mg/ml). The n-6:n-3 ratio was also calculated.

### Biomarker analyses

Biomarkers known to be influenced by n-3 PUFA were analysed. Serum lipids were analysed using the I-LAB 650 Chemical Analyser (Instrumentation Laboratories, Warrington, Cheshire, UK). LDL cholesterol was calculated using the Friedewald formula (Total cholesterol – HDL – (triglycerides/2.2)). The I-LAB 650 Chemical Analyser was also used to measure high-sensitivity C-reactive protein (hsCRP) in serum samples. The inflammatory markers interleukin (IL)-5, IL-10, IL-1β, IL-6 and tumour necrosis factor-alpha (TNF-α) were measured using an immunoassay from Meso Scale Discovery (MSD, Meso Scale Diagnostics, LLC, Maryland, USA). Samples with concentrations of inflammatory markers below the lower limit of detection (LLOD) had the LLOD/√2 inputted. Samples of whole blood were analysed for glutathione peroxidase (GPx), a marker of oxidative stress, using the Ransal Assay (Randox, UK) on the I-LAB 650 Chemical Analyser. At the time of processing, an aliquot of plasma had 0.005% BHT to allow for analysis of 8-isoprostanes. The plasma samples were then purified using 8-isoprostane affinity sorbent (Cayman Chemical, Ann Arbor, MI, USA), and analysed using an 8-isoprostane ELISA kit supplied by Cayman Chemical.

### Dietary intake and general health

Participants completed a 24-h recall of all foods and beverages consumed in the previous 24 h at both baseline and post-intervention appointments. Dietary intakes were quantified using Nutritics software (Nutritics Ltd, 2018). Participants also completed a health and lifestyle questionnaire to provide information on general health, including supplement usage, personal medical history and smoking and alcohol drinking habits, as well as demographic and socioeconomic data. At each lunchtime meal, participants were asked if they had eaten any fish in addition to the study portion. If additional fish was eaten, details including the type and quantity eaten were obtained from the participant.

### Statistical analysis

Statistical analyses of the data were completed using SPSS (Statistical Package for Social Sciences, Version 24.0. SPSS UK Ltd., Chertsey, UK). All statistical analyses were completed per protocol, and also using intention to treat (ITT). For ITT analyses, baseline values of subjects lost to follow-up were entered as post-intervention values [44]. All data were tested for normality, and skewed data were log transformed to approximate normality. Descriptive analyses were performed, and all data are expressed as median and interquartile range (25th, 75th percentiles). Differences in baseline characteristics between the three intervention groups were analysed using one-way analysis of variance (ANOVA).

In the primary analysis, analysis of covariance (ANCOVA), with LSD for post hoc comparison controlling for age, BMI and baseline serum PUFA concentrations, was used to investigate the effect of fish intervention on serum PUFA concentrations in the cohort stratified by genotype. Furthermore, ANCOVA analyses, controlling for age, BMI and relevant baseline concentrations were conducted to assess the effect of intervention on biomarkers of oxidative stress, inflammation and on lipid profile. In secondary analyses, participants within each treatment group were assigned into one of two genotype groups and the ANCOVA was repeated for homozygous carriers of the rs3834458 major allele (TT, *n* = 21) and for carriers of the minor allele (Tdel or deldel, *n* = 28) to assess the effect of intervention within genotype.

## Results

**Fig. 1:**
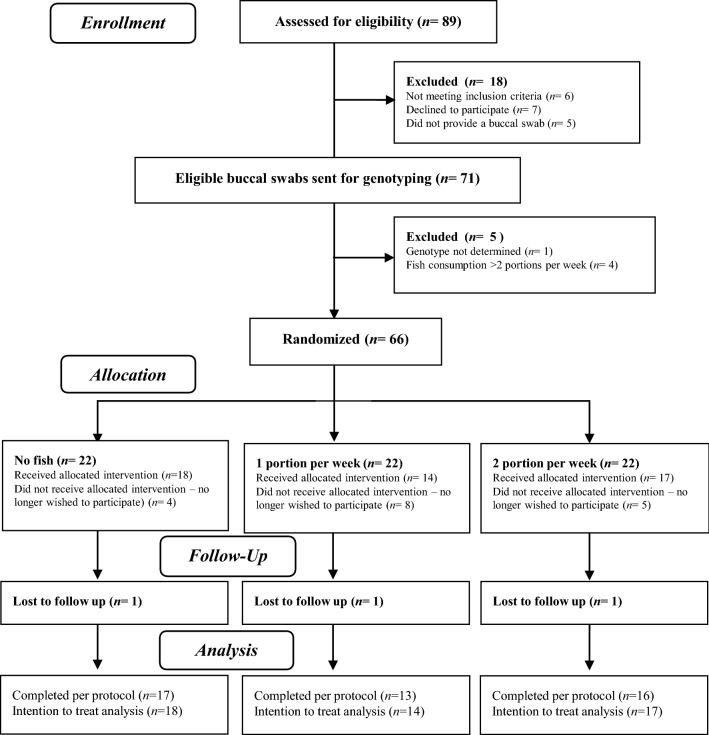
CONSORT flow diagram for study design

The CONSORT flow diagram outlines the number of people screened and recruited on to the study, and those who subsequently completed the intervention (Fig. 1). A total of 66 eligible participants for which buccal swabs were provided were randomised to 3 intervention groups. Of the 66 participants randomised, 49 completed a baseline appointment and began intervention. Three participants (1 from each intervention group) were dropouts from the study and were, therefore, absent at a follow-up post-intervention appointment (Fig. 1). Overall compliance was high (98%) as determined using the weight of lunchtime meals consumed. Compliance for each intervention group was 99%, 98% and 97% for no fish, 1 portion, and 2 portions groups, respectively. The median (IQR) age for this cohort was 23 (20, 30 years), with all participants being low consumers of fish (< 2 portions per week) at baseline. There were no significant differences in baseline characteristics between intervention groups (Table 1). *FADS2* rs3834458 was in Hardy–Weinberg equilibrium (*χ*^*2*^* p* value = 0.66). Genotype distribution among the intervention groups for rs3834458 is shown in Table 1.

**Table 1:** Characteristics of iFish study participants at baseline for the whole cohort, and according to intervention group

	Whole group (*n* = 49)	No fish (*n* = 18)	1 portion (*n* = 14)	2 portions (*n* = 17)	*P*-value*
Age (years)	23.0 (20.0, 30.0)	25.5 (21.0, 33.0)	23.0 (19.0, 31.8)	22.0 (20.0, 25.0)	0.766
Weight (kg)	62.7 (57.1, 74.3)	68.0 (55.9, 75.2)	61.0 (55.3, 69.4)	64.2 (57.3, 74.1)	0.513
Height (m)	1.67 (1.63, 1.70)	1.64 (1.62, 1.71)	1.67 (1.62, 1.69)	1.67 (1.64, 1.71)	0.676
BMI (kg/m2)	22.7 (20.7, 26.4)	23.5 (21.4, 26.9)	21.9 (20.3, 24.3)	23.3 (20.2, 25.5)	0.394
Fish consumption (portions/wk)	1.00 (0.50, 1.00)	0.75 (0.00, 1.00)	1.00 (0.50, 1.25)	1.00 (0.50, 1.00)	0.516
*Consumes alcohol*					
Yes	41 (83.7)	17 (94.4)	10 (71.4)	14 (82.4)	0.214
No	8 (16.3)	1 (5.6)	4 (28.6)	3 (17.6)	
*Smoker*					
Yes	5.0 (10.2)	2 (11.1)	3 (21.4)	0 (0)	0.144
No	44 (89.8)	16 (88.9)	11 (78.6)	17 (100)	
*Education*					
Secondary education	18 (36.7)	7 (38.9)	5 (35.7)	6 (35.3)	
FE college	5 (10.2)	0 (0.0)	3 (21.4)	2 (11.8)	
Undergraduate (BSc)	11 (22.4)	3 (16.7)	4 (28.6)	4 (23.5)	0.257
Postgraduate (MSc, PhD)	12 (24.5)	6 (33.3)	1 (7.1)	5 (29.4)	
Other	2 (4.1)	2 (11.1)	0 (0.0)	0 (0.0)	
*Employment*					
Work part time	11 (22.4)	1 (5.6)	5 (35.7)	5 (29.4)	
Work full time	8 (16.3)	2 (11.1)	2 (14.3)	4 (23.5)	0.141
Student	30 (61.2)	15 (83.3)	7 (50.0)	8 (27.1)	
*Genotype (n)*					
TT	21 (42.9)	8 (44.4)	7 (50.0)	6 (35.3)	
T/del	22 (44.9)	8 (44.4)	6 (42.9)	8 (47.1)	0.884
del	6 (12.2)	2 (11.1)	1 (7.1)	3 (17.6)	
*Baseline PUFA concentrations*					
LA (mg/ml)	0.263 (0.233, 0.304)	0.247 (0.212, 0.305)	0.258 (0.224, 0.310)	0.273 (0.253, 0.301)	0.559
ALA (mg/ml)	0.012 (0.012, 0.014)	0.012 (0.011, 0.013)	0.012 (0.012, 0.013)	0.013 (0.012, 0.014)	0.142
AA (mg/ml)	0.066 (0.054, 0.077)	0.067 (0.052, 0.075)	0.062 (0.052, 0.076)	0.070 (0.056, 0.082)	0.778
EPA (mg/ml)	0.012 (0.011, 0.014)	0.012 (0.011, 0.015)	0.012 (0.010, 0.013)	0.013 (0.011, 0.014)	0.108
DHA (mg/ml)	0.021 (0.018, 0.025)	0.024 (0.018, 0.026)	0.020 (0.017, 0.024)	0.020 (0.018, 0.026)	0.485
Total n-6 (mg/ml)	0.324 (0.295, 0.375)	0.318 (0.271, 0.366)	0.312 (0.286, 0.374)	0.343 (0.310, 0.375)	0.563
Total n-3 (mg/ml)	0.045 (0.041, 0.052)	0.049 (0.041, 0.053)	0.042 (0.040, 0.048)	0.046 (0.042, 0.053)	0.232
n-6:n-3 ratio	6.979 (6.457, 7.810)	6.579 (5.688, 7.279)	7.342 (6.554, 8.181)	7.174 (6.558, 7.916)	0.126
AA:LA ratio	0.255 (0.217, 0.295)	0.262 (0.216, 0.307)	0.257 (0.216, 0.284)	0.247 (0.216, 0.292)	0.843
EPA:ALA ratio	0.990 (0.909, 1.098)	0.991 (0.930, 1.145)	0.974 (0.839, 1.012)	0.994 (0.906, 1.114)	0.257
DHA:ALA ratio	1.696 (1.456, 2.044)	1.778 (1.575, 2.115)	1.553 (1.445, 1.895)	1.696 (1.340, 2.049)	0.335

Data are median (IQR), where IQR is the 25th and 75th centiles; or n (%) where appropriate; *BMI* body mass index, *FE* *college* Further education college, *LA* linoleic acid, *ALA* α-linolenic acid, *AA* arachidonic acid, *EPA* eicosapentaenoic acid, *DHA* docosahexaenoic acid

*p-value for significant difference between intervention groups at baseline as determined using ANOVA, or Chi square as appropriate; *p* < 0.05 considered significant

Results from ITT and per protocol analyses did not differ and ITT analysis is reported. The effect of intervention on serum PUFA concentrations at week 8, adjusting for covariates (baseline PUFA concentration, baseline age, and baseline BMI), is shown in Table 2. Intervention with 2 portions of fish per week significantly increased concentrations of EPA, DHA and total n-3 PUFA compared to consumption of no fish or one portion per week (all *p* < 0.05). The n-6:n-3 ratio was significantly lower for those in the two portions per week group compared to those in the no fish (p = 0.002) and one portion (*p* = 0.012) groups. There was no significant difference in serum PUFA concentrations at post-intervention between those consuming no fish and those consuming 1 portion per week. There were no significant differences in n-6 PUFA (LA, AA, total n-6) concentrations between intervention groups consuming different amounts of fish. No significant effects of intervention were found on concentrations of biomarkers of oxidative stress, inflammation or lipid profile analysed in the current study (Table 3). Baseline values for these biomarkers are shown in Online Resource 1. Post-intervention dietary intake data indicate that there was no significant difference in dietary intake of n-3 PUFA between intervention groups (Online Resource 2), or between genotype groups (Online Resource 3).

**Table 2:** The effect of dietary intervention with fish on post-intervention serum PUFA concentrations

PUFA (mg/ml)	No fish (*n* = 18)	1 portion (*n* = 14)	2 portions (*n* = 17)	*P*-value	Partial Eta squared
LA	0.272 (0.232, 0.300)	0.261 (0.246, 0.322)	0.294 (0.258, 0.322)	0.377	0.044
ALA	0.013 (0.012, 0.014)	0.012 (0.012, 0.013)	0.013 (0.012, 0.015)	0.375	0.045
AA	0.065 (0.055, 0.075)	0.070 (0.058, 0.086)	0.069 (0.059, 0.079)	0.375	0.045
EPA	0.012 (0.011, 0.013)^a^	0.014 (0.011, 0.015)^a^	0.014 (0.013, 0.025)^b^	< 0.001	0.311
DHA	0.021 (0.016, 0.024)^a^	0.022 (0.020, 0.029)^a^	0.028 (0.024, 0.034)^b^	< 0.001	0.347
Total *n*-6	0.343 (0.295, 0.364)	0.329 (0.317, 0.406)	0.375 (0.318, 0.392)	0.396	0.042
Total *n*-3	0.045 (0.042, 0.052)^a^	0.046 (0.038, 0.054)^a^	0.058 (0.051, 0.068)^b^	< 0.001	0.348
*n*6:*n*3 ratio	7.284 (6.586, 8.122)^a^	7.388 (6.992, 7.963)^a^	6.199 (4.897, 7.071)^b^	0.003	0.234
AA:LA ratio	0.243 (0.217, 0.297)	0.241 (0.218, 0.278)	0.234 (0.214, 0.276)	0.411	0.040
EPA:ALA ratio	0.933 (0.846, 1.058)	1.056 (0.889, 1.201)	1.043 (0.981, 1.993)	0.416	0.040
DHA:ALA ratio	1.590 (1.345, 1.934)	1.823 (1.599, 2.451)	2.168 (1.689, 2.603)	0.345	0.048

Data are median (IQR), where IQR is the 25th and 75th centiles; PUFA: polyunsaturated fatty acids; LA: linoleic acid; ALA: α-linolenic acid; AA: arachidonic acid; EPA: eicosapentaenoic acid; DHA docosahexaenoic acid; total *n*-6: LA + AA; total *n*-3: ALA + EPA + DHA; ANCOVA for effect of intervention, adjusting for baseline PUFA, baseline age, baseline BMI; *p* < 0.05 considered significant; different superscript letters represent significant differences between groups (LSD post hoc tests); logged variables for those not normally distributed

**Table 3:** The effect of dietary intervention with fish on biomarker status at post-intervention

Biomarker	No fish (*n* = 18)	1 portion (*n* = 14)	2 portions (*n* = 17)	*P*-value	Partial Eta squared
*Lipids (mmol/L)*					
Trigs	0.753 (0.590, 0.890)	0.695 (0.478, 1.075)	0.625 (0.445, 0.828)	0.403	0.041
Total chol	4.300 (3.500, 4.625)	3.925 (3.500, 4.675)	4.200 (3.725, 4.500)	0.865	0.007
HDL	1.485 (1.353, 1.655)	1.405 (1.340, 1.470)	1.530 (1.335, 1.720)	0.902	0.005
LDL	2.300 (1.809, 2.688)	2.018 (1.896, 2.818)	2.147 (1.903, 2.751)	0.607	0.023
TC:HDL	2.632 (2.393, 2.943)	2.744 (2.518, 3.463)	2.685 (2.299, 2.971)	0.881	0.006
non-HDL	2.625 (2.205, 2.975)	2.440 (2.165, 3.325)	2.510 (2.140, 3.020)	0.777	0.012
*Inflammatory markers (pg/ml)*					
IL-5	4.074 (1.362, 11.164)	1.773 (1.027, 4.278)	2.412 (1.149, 5.125)	0.165	0.080
IL-10	0.312 (0.181, 0.636)	0.373 (0.153, 0.518)	0.506 (0.245, 0.700)	0.388	0.043
IL-1β	0.120 (0.071, 0.183)	0.101 (0.029, 0.157)	0.161 (0.125, 0.295)	0.641	0.020
IL-6	0.627 (0.337, 1.391)	0.619 (0.377, 0.805)	0.490 (0.365, 0.814)	0.679	0.018
TNF-α	2.634 (2.385, 3.391)	2.873 (1.488, 3.785)	2.933 (2.346, 3.624)	0.983	0.001
CRP (µg/dL)	127.500 (39.250, 392.750)	68.500 (32.000, 219.250)	75.000 (32.500, 281.500)	0.899	0.005
*Oxidative stress*					
GPx (U/L)	9563.250 (7907.875, 10,398.625)	8620.250 (6959.750, 10,439.625)	9655.500 (7697.705, 11,326.250)	0.343	0.048
8-iso (pg/nl)	44.666 (35.527, 65.207)	29.412 (22.026, 70.417)	37.762 (30.384, 49.739)	0.335	0.050

Data expressed as median (IQR), where IQR is the 25th and 75th centiles; Trigs: Triglycerides; Total chol: Total cholesterol; HDL: High Density Lipoprotein; LDL: Low Density Lipoprotein; TC: Total cholesterol; IL: Interleukin; TNF-α: Tumour necrosis factor-alpha; CRP: C-reactive protein; GPx: Glutathione peroxidase; 8-iso: 8-isoprostanes; ANCOVA: Analysis of covariance; ANCOVA for effect of intervention, adjusting for baseline age, BMI, baseline biomarker status); P-value ≤ 0.05 considered significant; different letters represent significant differences from each other from LSD post hoc; logged variables for those not normally distributed

Following secondary analyses, two-way ANCOVA showed no interaction with genotype when investigating effects of intervention on PUFA. The effect of intervention within genotype was investigated (Table 4). Homozygous carriers of the major allele (TT), who consumed two portions of fish per week had significantly higher concentrations of EPA (*p *= 0.013), DHA (*p* = 0.001) and total *n*-3 PUFA (*p* = 0.014) compared to those in the no fish group. Post-intervention AA concentrations were significantly higher in the no fish group compared to the 1 portion of fish group (*p* = 0.016). The major allele homozygous genotype (TT) showed no significant effect of intervention on the *n*-6:*n*-3 ratio. Carriers of the minor allele (Tdel or deldel), who consumed two portions of fish per week had significantly higher total *n*-3 PUFA concentrations (*p* = 0.013) compared to those in the no fish group, but in contrast to the TT carriers, there was no significant increase in EPA or DHA concentrations. Furthermore, the minor allele carriers showed no significant effect of intervention on *n*-6 PUFA concentrations, or on the *n*-6:*n*-3 ratio. Comparison of post-intervention serum PUFA concentrations between homozygous carriers of the major allele (TT) and carriers of the minor allele (Tdel, deldel) is shown in Online Resource 4. Those who were carriers of the minor allele (Tdel, deldel) had significantly lower serum AA concentrations (*p* = 0.027) and a lower AA:LA ratio (*p* = 0.001) compared to major allele homozygotes (TT). No differences between homozygous carriers of the major allele (TT) and carriers of the minor allele (Tdel, deldel) were seen for any other PUFA. When comparison of post-intervention serum PUFA concentrations between homozygous carriers of the major allele (TT) and carriers of the minor allele (Tdel, deldel) was examined according to intervention group, higher ratios of AA:LA (*p* = 0.042) and DHA:ALA (*p* = 0.030) were found in major allele homozygotes (TT) for those consuming 2 portions of fish per week (Online Resource 5).

**Table 4:** The effect of intervention with fish on post-intervention serum PUFA concentrations according to *FADS2* rs3834458 genotype

PUFA (mg/ml)	TT (homozygotes of the major allele)					Tdel, deldel (carriers of the minor allele)				
	No fish (*n* = 8)	1 portion (*n* = 7)	2 portions (*n* = 6)	*P*-value	Partial Eta squared	No fish (*n* = 10)	1 portion (*n* = 7)	2 portions (*n* = 11)	*P*-value	Partial Eta squared
LA	0.256 (0.226, 0.283)	0.263 (0.246, 0.286)	0.285 (0.252, 0.310)	0.073	0.295	0.295 (0.238, 0.308)	0.258 (0.244, 0.347)	0.310 (0.263, 0.341)	0.760	0.025
ALA	0.013 (0.012, 0.015)	0.012 (0.012, 0.013)	0.012 (0.011, 0.013)	0.851	0.021	0.013 (0.012, 0.014)	0.012 (0.011, 0.013)	0.013 (0.013, 0.016)	0.206	0.134
AA	0.074 (0.058, 0.077)^a^	0.072 (0.064, 0.086)^b^	0.075 (0.068, 0.082)^ab^	0.049	0.331	0.061 (0.054, 0.072)	0.063 (0.040, 0.086)	0.068 (0.057, 0.077)	0.975	0.002
EPA	0.012 (0.011, 0.015)^a^	0.014 (0.012, 0.015)^a^	0.017 (0.014, 0.027)^b^	0.033	0.365	0.012 (0.009, 0.013)	0.014 (0.010, 0.015)	0.014 (0.013, 0.025)	0.055	0.231
DHA	0.021 (0.016, 0.026)^a^	0.022 (0.021, 0.026)^a^	0.032 (0.027, 0.036)^b^	0.002	0.551	0.021 (0.016, 0.023)	0.021 (0.018, 0.032)	0.027 (0.022, 0.030)	0.072	0.213
Total n-6	0.329 (0.293, 0.360)	0.332 (0.324, 0.354)	0.366 (0.320, 0.392)	0.057	0.318	0.350 (0.288, 0.379)	0.321 (0.284, 0.429)	0.378 (0.023, 0.030)	0.821	0.018
Total n-3	0.048 (0.042, 0.052)^a^	0.047 (0.043, 0.054)^ab^	0.061 (0.053, 0.076)^b^	0.043	0.342	0.045 (0.039, 0.050)^a^	0.044 (0.036, 0.055)^ab^	0.054 (0.050, 0.064)^b^	0.037	0.259
n6:n3 ratio	6.860 (6.287, 8.115)	7.319 (7.286, 7.527)	5.624 (4.948, 6.420)	0.090	0.274	7.570 (7.222, 8.323)	7.838 (6.110, 8.268)	6.617 (4.720, 7.085)	0.102	0.187
AA:LA ratio	0.267 (0.235, 0.305)	0.273 (0.233, 0.328)	0.276 (0.249, 0.281)	0.715	0.044	0.232 (0.191, 0.286)	0.223 (0.187, 0.274)	0.225 (0.186, 0.258)	0.660	0.037
EPA:ALA ratio	0.945 (0.822, 1.283)	1.053 (0.951, 1.153)	0.276 (0.249, 0.281)	0.850	0.021	0.922 (0.674, 1.005)	1.059 (0.855, 1.342)	1.000 (0.943, 1.991)	0.359	0.089
DHA:ALA ratio	1.590 (1.266, 2.215)	1.820 (1.637, 2.074)	2.570 (2.135, 3.018)	0.690	0.048	1.607 (1.344, 1.842)	2.104 (1.483, 2.629)	1.900 (1.663, 2.523)	0.279	0.110

Data expressed as median (IQR), where IQR is the 25th and 75th centiles; ANCOVA for effect of intervention, adjusting for baseline PUFA, baseline age, baseline *BMI*; *P* value ≤ 0.05 considered significant; different letters represent significant differences from each other from LSD post hoc; logged variables for those not normally distributed

## Discussion

To our knowledge, this is the first RCT which investigated the effects of increasing fish consumption on serum PUFA concentrations taking account of FADS genotype. Findings from this study indicate that consumption of two portions of fish per week significantly increases serum total PUFA concentrations of EPA, DHA and total *n*-3 compared to those consuming no fish or 1 portion per week when allowing for a major confounding factor of genetic variation in FADS2 (rs3834458, T/deletion). There was also a significant decrease in the *n*-6:*n*-3 ratio for those in the 2 portion group compared to the other intervention groups.

The current UK and USA dietary recommendations are to consume between two to three portions of fish per week, with at least one of these being oily fish [45, 46]. This study supports these recommendations as two portions of fish per week significantly increased *n*-3 LCPUFA status over 8 weeks in those who are habitually low fish consumers. Our findings confirm those of Miles et al*.* [34] who reported an increase in LCPUFA status in pregnant women consuming 2 portions of fish per week who were not habitual consumers of oily fish. Previous studies investigating the influence of fish consumption on PUFA status have not consistently found positive associations between fish intake and PUFA concentrations. Some studies have concluded that fish intake is not associated with *n*-3 PUFA status in pregnant women [9, 33] and non-pregnant populations [32]. In studies of pregnant women, the lack of an association was suggested to be owing to the increased transfer of *n*-3 LCPUFA to the developing foetus in the third trimester [9, 33]. In an observational study in non-pregnant adults, it was suggested that the metabolism of EPA and DHA from different fish oils may vary with fatty fish influencing biological LCPUFA status, but consumption of lean fish not having an influence on status. Also, a high dietary intake of *n*-6 LCPUFA may result in a decrease in *n*-3 LCPUFA absorption owing to competition for enzymes [32]. None of these studies have taken account of FADS genotype, which may have influenced associations. Addressing the disparities amongst studies, we stratified intervention groups by FADS genotype, and thus controlled for the possibility of genetic confounding. We found that higher fish consumption resulted in increased serum *n*-3 PUFA concentrations. Given the importance of *n*-3 LCPUFA for the developing foetus during pregnancy, all pregnant women or those of childbearing age would benefit from consumption of long-chain n-3 PUFA, as reflected in current FDA guidelines for fish consumption [46].

The decrease in the *n*-6:*n*-3 ratio in this study is also of interest. This ratio is regarded as a biological marker with a higher *n*-6:*n*-3 ratio associated with increased risk of obesity [47], cardiovascular disease and inflammatory diseases [48]. Also, an increased maternal *n*-6:*n*-3 fatty acid ratio is associated with poorer child development [49]. The current study reports a significant decrease in *n*-6:*n*-3 ratio from 7.174 to 6.199 in those consuming two portions of fish per week. A ratio of 3 to 4 has been suggested to be potentially beneficial in preventing early neurodegeneration, cancer and cardiovascular disease [50]. We hypothesise that with greater consumption of fish the *n*-6:*n*-3 ratio may decrease further.

N-3 LCPUFA are associated with reduced inflammation [51] and a lowering of lipid profile, specifically triglycerides and low-density lipoprotein [52]. As fish is a rich source of LCPUFA, it has been suggested that an increase in fish consumption may be beneficial for inflammation [37], oxidative stress [38] and lipid profile [39]. However, despite increased *n*-3 LCPUFA and decreased *n*-6:*n*-3 ratios with 2 portions of fish consumed, the current study did not detect any significant differences in any of the biomarkers analysed between groups. The absence of an effect of fish consumption on these biomarkers may be owing to the inclusion of healthy participants and small sample size, as the study was not powered to determine an effect on biomarkers of inflammation, oxidative stress and lipids. Previous studies which have reported an association between fish and lipid profile and inflammatory markers have been observational studies using self-reported intake of fish [37, 38]. This approach may result in fish intake reported being over or underestimated compared to what was actually consumed. Some RCT trials have investigated the influence of fish on lipid profile; however, these were powered to investigate the effect of fish consumption on lipid markers [39], and may explain the discrepancies between these findings and those reported in the current analysis. In the current study, it may be the case that greater fish consumption may increase circulating concentrations of *n*-3, and thereby lower the ratio further to a ratio observed to be beneficial. This effect in turn may impact on biomarkers of inflammation, oxidative stress and lipids.

Results of our secondary analysis found there was no interaction between fish intervention and genotype. The lack of a fish intervention × genotype interaction may be owing to insufficient statistical power to test this. Response to fish intake, however, seemed to differ within the genotype groups. Participants in the two portions of fish group carrying the minor allele had significantly higher total *n*-3 PUFA concentrations post-intervention compared to those in the no fish group. Major allele homozygotes in the two portion per week group had higher serum total *n*-3 PUFA, EPA and DHA concentrations following the intervention period compared to those in the no fish group. Therefore, increased fish intake was found to increase serum *n*-3 PUFA concentrations in both genotype groups, albeit this increase was more pronounced in individuals homozygous for the major allele. These findings suggest that consuming 2 portions of fish per week may result in higher circulating PUFA concentrations depending on genotype; however, further research is needed to confirm this possibility. At post-intervention, carriers of the minor allele (Tdel, deldel) had significantly lower serum AA concentrations and a lower AA:LA ratio compared to major allele homozygotes. These data suggest that endogenous synthesis of AA is not as efficient in those who are minor allele carriers, a finding which has been shown previously in other cohorts [18, 23, 53]. No differences in post-intervention concentrations of the *n*-3 PUFA EPA or DHA were found for major allele homozygotes (TT) compared to carriers of the minor allele (Tdel, deldel) and is in agreement with previous studies which suggest DHA may be less influenced by variation in FADS genotype [18, 53]. When examined according to intervention group, those in the 2 portions of fish per week group and who were homozygous major allele carriers (TT) had a higher AA:LA and DHA:ALA ratio compared to minor allele carriers (Tdel, deldel). This finding is likely owing to more efficient endogenous synthesis of PUFA in major allele homozygotes, and in the case of DHA:ALA, the increased consumption of preformed DHA from 2 portions of fish per week has resulted in a higher ratio.

The presence of the minor allele has been linked to decreased synthesis of LCPUFA via the elongation pathway [16, 41]. Some intervention studies have investigated the influence of PUFA supplementation on biological PUFA status according to FADS genotype, and reported that n-3 PUFA supplements increase DHA in minor allele carriers [54]. A previous intervention study investigated whether fish oil supplementation was associated with desaturase enzyme activity according to FADS genotype. Following supplementation there was an association between FADS genotype and increased D5D activity, suggesting n-3 PUFA supplementation may influence PUFA metabolism when genetic variation is present [55]. Dietary intake data collected at post-intervention appointments show that there was no significant difference in dietary intake of PUFA between the genotype groups (Online Resource 3). Major allele homozygotes may synthesise LCPUFA more efficiently in the body from precursor molecules, which coupled with increased dietary intake could explain the increased biological concentrations at post-intervention. Individuals with certain genotypes, particularly minor allele carriers, may respond differently to fish consumption and thus further research is needed to investigate this possibility. The *n*-6 dietary intake of participants in the current study at post-intervention was 6.92 g/day and 7.87 g/day for those with the Tdel and deldel genotypes, respectively. This intake is lower than previously reported n-6 intakes of 8.6 g/day in the UK, and the European average of 11.9 g/day [56]. The lower intakes reported in the current study may in part be owing to the dietary assessment method used. A 24-h recall was used in the current study to record habitual food intake data; however, this is a limitation of the study as a food frequency questionnaire (FFQ) or weighed food dairy may have provided more robust dietary data. It is also possible that the dietary analysis software used did not contain detailed information on PUFA composition of foods.

Further limitations of the study include only screening for one particular genotype (rs3834458). Other FADS SNPs not in linkage disequilibrium with rs3834458 could be considered. Genetic variation in the ELOVL genes which encode the elongase enzymes has been found to influence PUFA status [55] and therefore should be considered in future studies. PUFA analysis was completed in serum, which is considered to be reflective of dietary intake in approximately the last 24 h before sampling, however, measurement of PUFA in serum lipoproteins or erythrocyte membranes may have better reflected the intake over the intervention period. The sample size of the current study was not powered to investigate the influence of fish consumption on lipids or markers of inflammation and oxidative stress. Also, high trans-fatty acid (TFA) and saturated fatty acid status have been shown to mask some effects of PUFAs on oxidative stress, inflammation and lipids [57] but were not taken into account in the current analysis. This study also has many strengths. To our knowledge, this is the first intervention study investigating the influence of fish consumption on serum n-3 PUFA concentrations while allowing for a major confounding factor of genetic variation in FADS2. Also, compliance was high for the intervention study. GC–MS is regarded as the gold standard method for PUFA analysis which is a further strength of the study.

Various factors should be considered in future work such as the maximum amount of fish given, as higher consumption of fish may further increase PUFA status and warrants further investigation. The intervention period for the current study was 8 weeks as this was deemed suitable to see a change in PUFA status; however, it is possible that a longer intervention period may have allowed further changes in PUFA status to occur. In this study we chose to stratify by rs3834458 to account for the effect of genotype during randomisation; however, future studies should consider screening participants for a particular genotype and intervening with these people to see how fish consumption influenced PUFA in that particular genotype.

## Conclusion

This is the first intervention study to show that consumption of 2 portions of fish per week resulted in a significant increase in serum *n*-3 PUFA concentrations and a decrease in the *n*-6:*n*-3 ratio compared to consumption of no fish or one portion of fish in women of childbearing age while stratifying by FADS genotype. This study supports the current international public health guidelines to consume 2 portions of fish per week. Fish consumption was found to have no effect on markers of inflammation or oxidative stress in the current study; however, greater fish intake may be necessary to influence these biomarkers. This study, for the first time, stratified by FADS genotype during randomisation and, therefore, takes account of confounding from genotype so that the influence of fish consumption was not biased by any genetic effect. Future research in this area should investigate the effects of higher intakes of fish on PUFA status taking into consideration FADS genotype.

## Electronic supplementary material

Below is the link to the electronic supplementary material.

Supplementary file1 (DOCX 40 kb)

## Abbreviations

AA: Arachidonic acid
ALA: α-Linolenic acid
ANCOVA: Analysis of covariance
BMI: Body mass index
DHA: Docosahexaenoic acid
EPA: Eicosapentaenoic acid
FADS: Fatty acid desaturase
GCMS: Gas chromatography mass spectrometry
HISU: Human intervention studies unit
IL: Interleukin
LA: Linoleic acid
LCPUFA: Long-chain polyunsaturated fatty acids
PUFA: Polyunsaturated fatty acid
RCT: Randomised controlled trial
SPSS: Statistical package for social sciences
UREC: University research ethics committee
