# Supplementary material for: The influence of fish consumption on serum n-3 polyunsaturated fatty acid (PUFA) concentrations in women of childbearing age: a randomised controlled trial (the iFish Study)
Source: Eur J Nutr. 2020 Jul 28;60(3):1415–27. doi: 10.1007/s00394-020-02326-w (PMC7987591; doi:10.1007/s00394-020-02326-w)
Supplement: Supplementary file 1 — Supplementary file1 (DOCX 40 kb) [file 394_2020_2326_MOESM1_ESM.docx]

**The influence of fish consumption on serum n-3 polyunsaturated fatty acid (PUFA) concentrations in women of child bearing age - a randomised controlled trial (the iFish Study)**

Marie C Conway^1^, Emeir M McSorley^1^, Maria S Mulhern^1^, Toni Spence^1^, Edwin van Wijngaarden^2^, Gene E Watson^2^, Karin Wahlberg^3^, Daniela Pineda^3^, Karin Broberg^3,4^, Barry W Hyland^5^, Diego F Cobice^5^, JJ Strain^1^, and Alison J Yeates^1*^

^1^Nutrition Innovation Centre for Food and Health (NICHE), Ulster University, Coleraine, Northern Ireland, ^2^School of Medicine and Dentistry, University of Rochester, Rochester, NY, ^3^The Laboratory of Medicine, Division of Occupational and Environmental Medicine, Lund University, Lund, Sweden, ^4^Institute of Environmental Medicine, Karolinska Institutet, Stockholm, Sweden, ^5^Mass Spectrometry Centre, Biomedical Sciences Research Institute (BMSRI), Ulster University, Coleraine, Northern Ireland, UK.

*Correspondence to Alison J Yeates, Nutrition Innovation Centre for Food and Health (NICHE), School of Biomedical Sciences, Ulster University, Cromore Road, Coleraine, Northern Ireland, BT52 1SA, United Kingdom. Tel.: +44-28-7012-3147. Email: [a.yeates@ulster.ac.uk](mailto:a.yeates@ulster.ac.uk)

| **Online Resource 1:** Baseline biomarker status of iFish participants according to intervention group | | | | |
| --- | --- | --- | --- | --- |
| **Biomarker** | **No fish**  **(*n*=18)** | **1 portion**  **(*n*=14)** | **2 portions**  **(*n*=17)** | ***P*-value** |
| ***Lipids (mmol/L)*** |  |  |  |  |
| **Trigs** | 0.698 (0.548, 0.939) | 0.790 (0.591, 1.031) | 0.635 (0.455, 0.895) | 0.570 |
| **Total chol** | 4.050 (3.338, 4.650) | 3.975 (3.475, 4.763) | 4.100 (3.625, 4.775) | 0.884 |
| **HDL** | 1.530 (1.483, 1.763) | 1.515 (1.330, 1.628) | 1.630 (1.490, 1.800) | 0.183 |
| **LDL** | 1.980 (1.619, 1.447) | 2.093 (1.652, 2.679) | 2.047 (1.661, 2.627) | 0.950 |
| **TC:HDL** | 2.383 (2.203, 2.809) | 2.664 (2.305, 3.232) | 2.487 (2.206, 2.879) | 0.494 |
| **non-HDL** | 2.265 (1.850, 2.833) | 2.360 (1.913, 3.313) | 2.420 (1.980, 2.900) | 0.875 |
| ***Inflammatory markers (pg/ml)*** |  |  |  |  |
| **IL-5** | 0.439 (0.167, 1.576) | 0.418 (0.281, 1.145) | 0.625 (0.418, 0.910) | 0.923 |
| **IL-10** | 0.176 (0.022, 0.502) | 0.029 (0.021, 0.184) | 0.261 (0.061, 0.531) | 0.250 |
| **IL-1β** | 0.262 (0.173, 0.319) | 0.234 (0.174, 0.276) | 0.276 (0.200, 0.317) | 0.162 |
| **IL-6** | 0.609 (0.369, 0.975) | 0.551 (0.349, 0.675) | 0.535 (0.358, 1.129) | 0.273 |
| **TNF-α** | 4.393 (3.558, 5.005) | 3.381 (2.607, 4.043) | 4.097 (3.504, 4.639) | 0.079 |
| **CRP (µg/dL)** | 128.500 (57.500, 414.500) | 77.000 (24.750, 233.250) | 57.000 (30.500, 363.000) | 0.393 |
| ***Oxidative stress*** |  |  |  |  |
| **GPx (U/L)** | 10588.250 (9153.250, 11562.000) | 10557.500 (7728.500, 11690.125) | 9922.000 (9409.500, 11746.500) | 0.760 |
| **8-iso (pg/nl)** | 40.998 (31.796, 56.708) | 44.248 (30.056, 60.615) | 33.880 (23.078, 55.210) | 0.359 |
| *Data expressed as median (IQR), where IQR is the 25^th^ and 75^th^ centile; Trigs: Triglycerides; Total chol: Total cholesterol; HDL: High Density Lipoprotein; LDL: Low Density Lipoprotein; TC: Total cholesterol; IL: Interleukin; TNF-α: Tumour necrosis factor-alpha; CRP: C-reactive protein; GPx: Glutathione peroxidase;8-iso: 8-isoprostanes; *p-value for significant difference between intervention groups at baseline as determined using ANOVA, p<0.05 considered significant; P-value ≤0.05 considered significant; different letters represent significant differences from each other from LSD post hoc; logged variables for those not normally distributed* | | | | |

| **Online Resource 2:** Dietary intake of macronutrients and PUFA at post intervention in each intervention group | | | | |
| --- | --- | --- | --- | --- |
|  | **No fish**  **(n=18)** | **1 portion**  **(n=14)** | **2 portions**  **(n=17)** | ***P*-value** |
| **Energy (kcal/day)** | 1692.70 (1467.72, 2086.57)^ab^ | 1596.48 (1221.69, 1806.22)^a^ | 2003.69 (1597.40, 2268.98)^b^ | **0.030** |
| **Energy (kJ/day)** | 7104.93 (6171.54, 8718.33)^ab^ | 6597.47 (5122.39, 7589.87)^a^ | 8387.03 (6708.76, 9546.91)^b^ | **0.030** |
| **Protein (g/day)** | 75.60 (67.94, 100.78) | 79.62 (65.47, 89.52) | 88.18 (76.02, 102.08) | 0.133 |
| **Protein (%energy)** | 19.78 (17.28, 22.70) | 20.65 (16.68, 23.09) | 18.24 (15.53, 23.13) | 0.579 |
| **Fat (g/day)** | 66.96 (58.05, 85.25) | 58.02 (48.80, 67.22) | 78.04 (65.14, 96.41) | 0.083 |
| **Fat (%energy)** | 36.39 (32.05, 38.73) | 36.03 (31.81, 39.02) | 32.27 (32.02, 41.82) | 0.989 |
| **Carbohydrates (g/day)** | 194.17 (153.00, 215.31) | 170.87 (120.43, 208.41) | 198.45 (170.68, 266.63) | 0.054 |
| **Carbohydrates (%energy)** | 43.23 (41.32, 48.76) | 40.77 (38.07, 46.31) | 42.48 (37.37, 49.12) | 0.599 |
| **Saturated fat (g/day)** | 25.57 (19.46, 29.68)^a^ | 19.89 (11.67, 22.25)^b^ | 23.13 (20.67, 31.61)^ab^ | **0.036** |
| **Omega-6 (Total)** | 4.64 (3.00, 6.42)^a^ | 7.73 (4.90, 10.61)^ab^ | 9.38 (7.86, 11.48)^b^ | **0.036** |
| **Omega-3 (Total)** | 1.51 (0.81, 1.90) | 1.39 (0.49, 2.31) | 1.57 (1.00, 4.28) | 0.320 |
| **MUFA (g/day)** | 25.02 (17.60, 31.79) | 21.93 (15.12, 30.18) | 26.01 (22.93, 38.40) | 0.085 |
| **PUFA (g/day)** | 9.98 (7.60, 12.53) | 11.04 (6.59, 15.96) | 16.09 (12.10, 20.69) | 0.085 |
| **Cholesterol** | 200.69 (159.24, 359.15) | 144.94 (95.79, 270.75) | 179.83 (142.20, 290.43) | 0.216 |
|  |  |  |  |  |
| ***PUFA (g/day)*** |  |  |  |  |
| **C18:2 (LA)** | 1.78 (0.72, 3.83) | 2.28 (0.54, 4.03) | 1.41 (0.70, 2.57) | 0.515 |
| **C18:3 (ALA)** | 0.27 (0.06, 0.63) | 0.31 (0.06, 0.56) | 0.14 (0.05, 0.30) | 0.279 |
| **C20:4cn6 (AA)** | 0.02 (0.01, 0.05) | 0.01 (0.00, 0.04) | 0.02 (0.01, 0.09) | 0.578 |
| **C20:5cn3 (EPA)** | 0.01 (0.00, 0.02) | 0.00 (0.00, 0.01) | 0.01 (0.00, 0.01) | 0.286 |
| **C22:6cn3 (DHA)** | 0.01 (0.00, 0.04) | 0.00 (0.00, 0.01) | 0.04 (0.00, 0.06) | 0.445 |
| *Data expressed as median (IQR), where IQR is the 25^th^ and 75^th^ centile; PUFA: polyunsaturated fatty acids; LA: linoleic acid; ALA: α-linolenic acid; AA: arachidonic acid; EPA: eicosapentaenoic acid; DHA docosahexaenoic acid; total n-6: LA+AA; total n-3: ALA+EPA+DHA; ANOVA: Analysis of variance; ANOVA or differences between intervention groups; p­-value ≤0.05 considered significant* | | | | |

| **Online Resource 3:** Dietary intake of n-6 and n-3 polyunsaturated fatty acids (PUFA) in major allele (TT) and carriers of the minor allele (Tdel, deldel) at post intervention | | | |
| --- | --- | --- | --- |
| **PUFA (g/day)** | **TT (*n*=21)** | **Tdel, deldel (*n*=28)** | ***P*-value** |
| **Omega-6 (Total)** | 6.92 (4.33, 9.98) | 7.87 (4.06, 9.79) | 0.925 |
| **Omega-3 (Total)** | 1.42 (0.87, 2.23) | 1.56 (0.75, 2.52) | 0.770 |
| **PUFA Polyunsaturated Fat** | 11.42 (7.71, 15.35) | 12.81 (8.62, 18.38) | 0.345 |
| **C18:2 Linoleic Acid (LA)** | 0.64 (0.03, 1.38) | 1.06 (0.53, 2.00) | 0.070 |
| **C18:3 Linolenic Acid (ALA)** | 0.07 (0.02, 0.14) | 0.13 (0.05, 0.32) | 0.175 |
| **C20:4cn6 Arachidonic Acid (AA)** | 0.01 (0.01, 0.04) | 0.02 (0.01, 0.05) | 0.393 |
| **C20:5cn3 Eicosapentaenoic Acid (EPA)** | 0.00 (0.00, 0.01) | 0.01 (0.00, 0.02) | 0.150 |
| **C22:6cn3 Docosahexaenoic Acid (DHA)** | 0.00 (0.00, 0.04) | 0.01 (0.00, 0.04) | 0.867 |
| *Data expressed as median (IQR), where IQR is the 25^th^ and 75^th^ centile; PUFA: polyunsaturated fatty acids; LA: linoleic acid; ALA: α-linolenic acid; AA: arachidonic acid; EPA: eicosapentaenoic acid; DHA docosahexaenoic acid; logged variables for those not normally distributed; Independent sample t test for differences between TT and Tdel, deldel genotype groups; P­-value ≤0.05 considered significant* | | | |

| **Online Resource 4:** Differences in serum PUFA concentrations in major allele (TT) and carriers of the minor allele (Tdel, deldel) at post intervention | | | |  |
| --- | --- | --- | --- | --- |
| **PUFA (mg/ml)** | **TT (n=21)** | **Tdel, deldel (n=28)** | ***P*-value** |  |
| **LA** | 0.269 (0.245, 0.288) | 0.295 (0.255, 0.320) | 0.519 |  |
| **ALA** | 0.013 (0.012, 0.014) | 0.013 (0.012, 0.014) | 0.453 |  |
| **AA** | 0.074 (0.065, 0.081) | 0.063 (0.053, 0.072) | **0.027** |  |
| **EPA** | 0.014 (0.012, 0.016) | 0.013 (0.011, 0.015) | 0.383 |  |
| **DHA** | 0.024 (0.021, 0.029) | 0.023 (0.020, 0.028) | 0.576 |  |
| **Total n-6** | 0.339 (0.321, 0.364) | 0.350 (0.301, 0.397) | 0.970 |  |
| **Total n-3** | 0.052 (0.044, 0.057) | 0.050 (0.044, 0.055) | 0.617 |  |
| **n6:n3 ratio** | 7.053 (5.982, 7.481) | 7.263 (6.235, 7.958) | 0.546 |  |
| **AA:LA ratio** | 0.273 (0.235, 0.294) | 0.225 (0.189, 0.270) | **0.001** |  |
| **EPA:ALA ratio** | 1.053 (0.944, 1.618) | 0.985 (0.891, 1.153) | 0.293 |  |
| **DHA:ALA ratio** | 2.038 (1.590, 2.570) | 1.746 (1.508, 2.385) | 0.345 |  |
| *Data are median (IQR), where IQR is 25^th^, 75^th^ centile; PUFA: polyunsaturated fatty acids; LA: linoleic acid; ALA: α-linolenic acid; AA: arachidonic acid; EPA: eicosapentaenoic acid; DHA docosahexaenoic acid; total n-6: LA+AA; total n-3: ALA+EPA+DHA; logged variables for those not normally distributed; Independent sample t test for differences between TT and Tdel, deldel genotype groups; P-value ≤0.05 considered significant* | | | |  |
|  |  |  |  |  |

| **Online Resource 5:** Serum PUFA concentrations in major allele (TT) and carriers of the minor allele (Tdel, deldel) at post intervention according to intervention group | | | | | | | | | |
| --- | --- | --- | --- | --- | --- | --- | --- | --- | --- |
|  | **No fish (n=18)** | | | **1 portion (n=14)** | | | **2 portions (n=17)** | | |
| **PUFA (mg/ml)** | **TT (n=8)** | **Tdel, deldel (n=10)** | ***P*-value** | **TT (n=7)** | **Tdel, deldel (n=7)** | ***P*-value** | **TT (n=6)** | **Tdel, deldel (n=11)** | ***P*-value** |
| **LA** | 0.256 (0.226, 0.283) | 0.295 (0.238, 0.308) | 0.378 | 0.263 (0.246, 0.286) | 0.258 (0.244, 0.347) | 0.806 | 0.285 (0.252, 0.310) | 0.310 (0.263, 0.341) | 0.678 |
| **ALA** | 0.013 (0.012, 0.015) | 0.013 (0.012, 0.014) | 0.609 | 0.012 (0.012, 0.013) | 0.012 (0.011, 0.013) | 0.798 | 0.012 (0.011, 0.013) | 0.013 (0.013, 0.016) | 0.057 |
| **AA** | 0.074 (0.058, 0.077) | 0.061 (0.054, 0.072) | 0.365 | 0.072 (0.064, 0.086) | 0.063 (0.040, 0.086) | 0.229 | 0.075 (0.068, 0.082) | 0.068 (0.057, 0.077) | 0.071 |
| **EPA** | 0.012 (0.011, 0.015) | 0.012 (0.009, 0.013) | 0.191 | 0.014 (0.012, 0.015) | 0.014 (0.010, 0.015) | 0.345 | 0.017 (0.014,0.027) | 0.014 (0.013, 0.025) | 0.612 |
| **DHA** | 0.021 (0.016, 0.026) | 0.021 (0.016, 0.023) | 0.840 | 0.022 (0.021, 0.026) | 0.021 (0.018, 0.032) | 0.951 | 0.032 (0.027, 0.036) | 0.027 (0.023, 0.030) | 0.177 |
| **Total n-6** | 0.329 (0.293, 0.360) | 0.350 (0.288, 0.379) | 0.604 | 0.332 (0.324, 0.354) | 0.321 (0.284, 0.429) | 0.614 | 0.366 (0.320, 0.392) | 0.378 (0.313, 0.401) | 0.987 |
| **Total n-3** | 0.048 (0.042, 0.052) | 0.045 (0.039, 0.050) | 0.580 | 0.047 (0.043, 0.054) | 0.044 (0.036, 0.055) | 0.611 | 0.061 (0.053, 0.076) | 0.054 (0.050, 0.064) | 0.463 |
| **n6:n3 ratio** | 6.860 (6.287, 8.115) | 7.570 (7.222, 8.323) | 0.403 | 7.319 (7.286, 7.527) | 7.838 (6.110, 8.268) | 0.982 | 5.624 (4.948, 6.420) | 6.617 (4.720, 7.085) | 0.386 |
| **AA:LA ratio** | 0.267 (0.235, 0.306) | 0.232 (0.191, 0.286) | 0.140 | 0.273 (0.234, 0.328) | 0.223 (0.187, 0.274) | 0.073 | 0.276 (0.249, 0.281) | 0.226 (0.186, 0.258) | **0.042** |
| **EPA:ALA ratio** | 0.945 (0.822, 1.283) | 0.922 (0.674, 1.006) | 0.203 | 1.053 (0.951, 1.153) | 1.059 (0.855, 1.342) | 0.938 | 1.524 (1.016, 2.185) | 1.000 (0.943, 1.991) | 0.320 |
| **DHA:ALA ratio** | 1.590 (1.266, 2.215) | 1.607 (1.344, 1.842) | 0.358 | 1.820 (1.637, 2.074) | 2.104 (1.483, 2.629) | 0.999 | 2.570 (2.135, 3.018) | 1.900 (1.663, 2.523) | **0.030** |
| *Data are median (IQR), where IQR is 25^th^, 75^th^ centile; PUFA: polyunsaturated fatty acids; LA: linoleic acid; ALA: α-linolenic acid; AA: arachidonic acid; EPA: eicosapentaenoic acid; DHA docosahexaenoic acid; total n-6: LA+AA; total n-3: ALA+EPA+DHA; logged variables for those not normally distributed; Independent sample t test for differences between TT and Tdel, deldel genotype groups; P-value ≤0.05 considered significant* | | | | | | | | | |
|  |  |  |  |  |  |  |  |  |  |
